# Transsphenoidal hypophysectomy for the treatment of hypersomatotropism secondary to a pituitary somatotroph adenoma in a dog

**DOI:** 10.1111/jvim.16929

**Published:** 2023-11-02

**Authors:** Matthew M. E. Steele, Jack S. Lawson, Christopher Scudder, Alice H. Watson, Nicola T. Z. Ho, Dylan Yaffy, Daniel Batchelor, Joe Fenn

**Affiliations:** ^1^ Department of Clinical Science and Services Royal Veterinary College Hatfield UK; ^2^ Department of Pathobiology and Population Sciences Royal Veterinary College Hatfield UK; ^3^ Department of Small Animal Clinical Sciences University of Liverpool Neston UK

**Keywords:** acromegaly, canine, endocrine, growth hormone, insulin‐like growth factor‐1, surgery

## Abstract

Pituitary‐dependent hypersomatotropism is rarely diagnosed in dogs and surgical treatment is not reported. A 6‐year‐10‐month male neutered Patterdale Terrier presented with polyuria, polydipsia, progressive pharyngeal stertor, excessive hair growth and widened facial features and paws. Serum insulin‐like growth factor‐1 concentration via radioimmunoassay was consistent with hypersomatotropism (1783 ng/mL). A pituitary mass was identified on magnetic resonance and computed tomography imaging. Six weeks later, glucosuria, starved hyperglycemia and serum fructosamine above the reference range (467.6 μmol/L, RI 177‐314) were documented, consistent with diabetes mellitus. Transsphenoidal hypophysectomy was performed under general anesthesia without complications. Pituitary histopathology identified an acidophil neoplasm, with positive immunostaining for growth hormone. Postoperatively, there was rapid resolution of clinical, biochemical and morphologic changes of hypersomatotropism with persistence of diabetes mellitus. This case demonstrates successful resolution of hypersomatotropism with ongoing diabetes mellitus in a dog after surgical treatment by transsphenoidal hypophysectomy.

**Abbreviations**
CT: computed tomography
DM: diabetes mellitus
GH: growth hormone
HS: hypersomatotropism
IGF‐1: insulin‐like growth factor 1
PRL: prolactin
PUPD: polyuria and polydipsia
TT4: total thyroxine

## 1 INTRODUCTION

Acromegaly or hypersomatotropism (HS), the condition of growth hormone (GH) excess, might result in morphologic changes and a state of insulin‐resistant diabetes mellitus (DM).^1^ Acromegaly as a result of a GH‐producing pituitary somatotroph adenoma is rare in dogs, with few reports of successful treatment.^2^, ^3^, ^4^ This case report describes the diagnosis, management, and outcome of a dog with acromegaly and DM associated with a pituitary adenoma treated surgically by transsphenoidal hypophysectomy.

## 2 CASE DESCRIPTION

**FIGURE 1:**
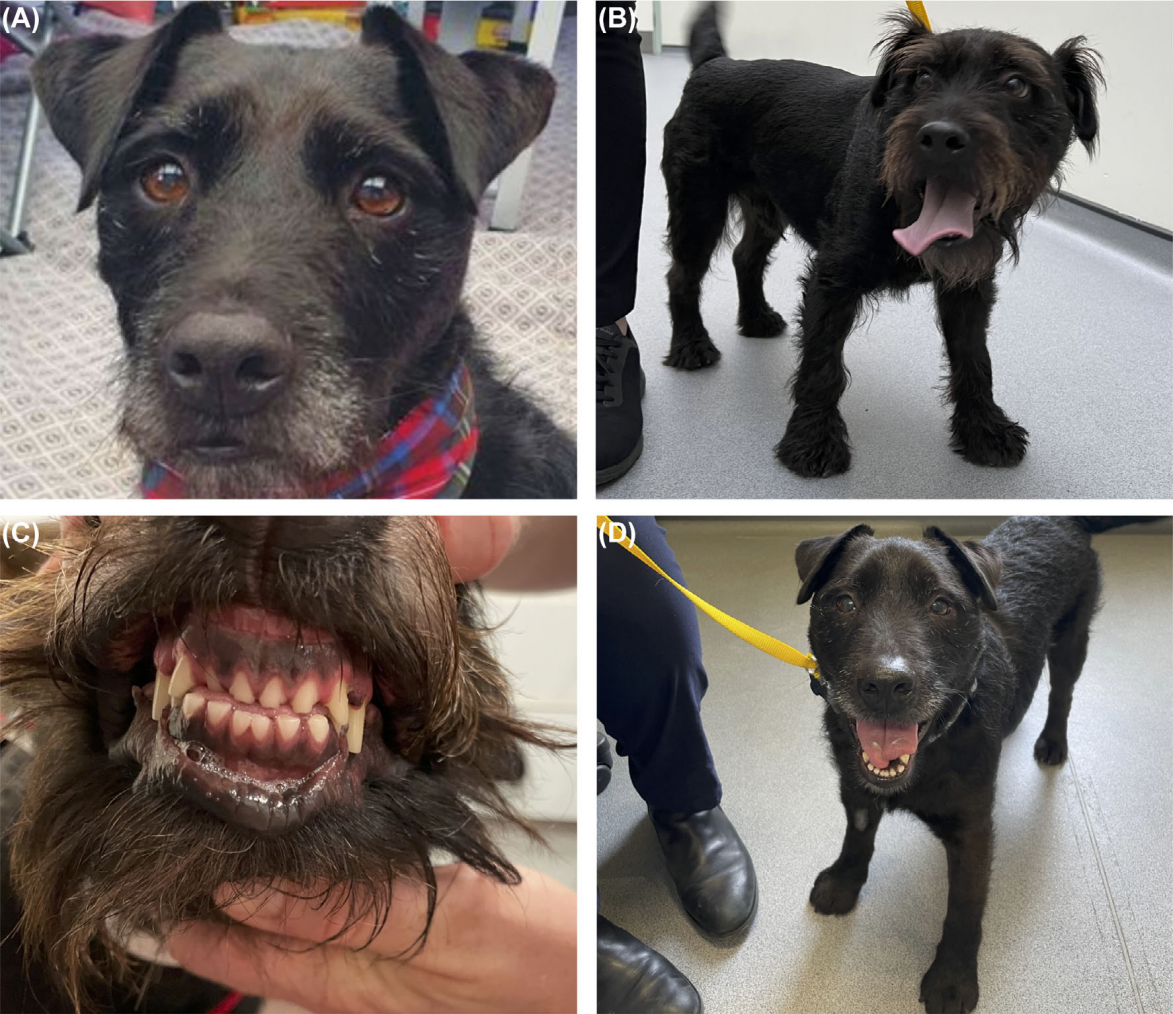
Photographs illustrating morphological changes in a 6‐year‐10‐month male neutered Patterdale Terrier with acromegaly. (A) Normal hair length before diagnosis. (B) Increased hair length and large paws noted by the owners at the time of diagnosis. (C) Prognathism and widened interdental spaces seen preoperatively. (D) Normalization of hair length seen at 11‐week postoperative reexamination.

A 6‐year‐10‐month male neutered Patterdale Terrier presented to a referral center (University of Liverpool, Small Animal Teaching Hospital) with a 3‐month history of polyuria and polydipsia (PUPD), weight gain, lethargy, increased coat length and progressive inspiratory stertor. Physical examination revealed broad facial features, widened paws and increased facial hair length (Figure 1). Interdental spaces were wide and there was a mild prognathia inferior (Figure 1). A mild pharyngeal stertor was evident. No other abnormalities were detected on physical examination.

Hematology and serum biochemistry findings at presentation are documented in Supplemental Tables 1 and 2. Urinalysis did not reveal abnormalities with no glucosuria present. Urine protein: creatinine ratio was 0.56 (RI < 0.5). Thyroid hormone tests were consistent with euthyroidism as serum total thyroxine (TT4) was 1.42 μg/dL (RI 0.39‐3.42 μg/dL; 18.3 nmol/L; RI 5‐44 nmol/L) and concurrent serum thyroid stimulating hormone was 0.118 ng/mL (RI 0.03‐0.50 ng/mL). A low dose dexamethasone suppression test was not consistent with hypercortisolism (serum basal cortisol 1.38 μg/dL; RI 1.01‐9.06 μg/dL; 38 nmol/L; RI 28‐250 nmol/L), 3 and 8 hour postdexamethasone serum cortisol were both <1.01 μg/dL (RI < 1.45 μg/dL; <27.9 nmol/L; RI < 40 nmol/L). Serum insulin‐like growth factor 1 (IGF‐1) concentration was 1783 ng/mL (>1000 ng/mL being consistent with a diagnosis of acromegaly, radioimmunoassay validated for use in dogs, Nationwide Specialist Laboratory Services Ltd, UK). Serum progesterone concentration was 0.7 nmol/L (<3 nmol/L consistent with anoestrus in a bitch).

**FIGURE 2:**
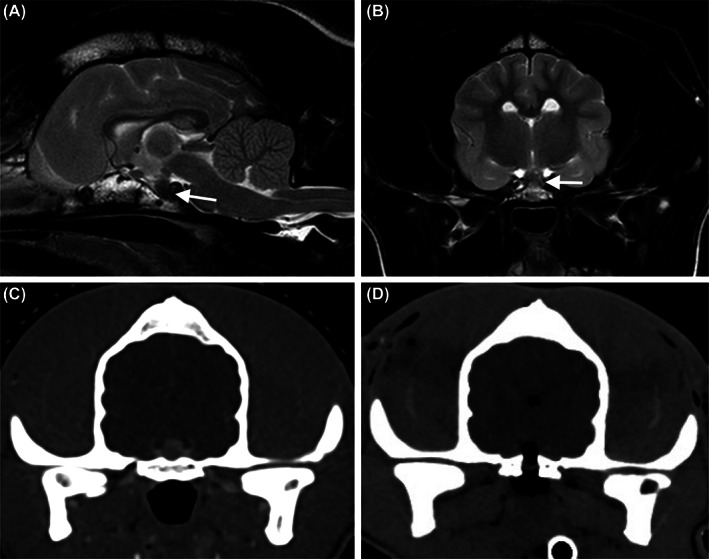
Sagittal (A) and transverse (B) T2‐weighted magnetic resonance imaging (MRI) image of the brain, showing a largely T2‐weighted hypointense, moderate enlargement of the pituitary gland, with no significant mass effect (white arrows). Transverse computed tomography (CT) reconstructions of the pituitary mass at the time of diagnosis (C—post contrast) and 15 minutes postoperatively under the same general anesthetic (D), demonstrating the absence of the previously present contrast‐enhancing pituitary mass. Postoperatively the surgical burr hole in the basisphenoid bone is evident, ventral to a radiolucent air‐filled pituitary fossa, with a small volume of air present in the third ventricle.

Magnetic resonance imaging (Ingenia Ambition 1.5 Tesla, Phillips, Amsterdam, Netherlands) of the head revealed a moderately enlarged pituitary gland (maximum height 6.4 mm), predominantly hypo‐ to isointense to surrounding gray matter on T2‐weighted images with no evidence of mass effect on the surrounding brain parenchyma (Figure 2). Computed tomography (CT; Aquilion Lightning 80, Canon Medical Systems, California, United States) of the head, thorax and abdomen demonstrated a moderately enlarged, uniformly contrast‐enhancing pituitary gland (Figure 2) as well as thickened soft palate/pharyngeal tissue and thickened cortices of the humerus, femur and tibia. There was mild hepatomegaly, and the remainder of the CT study did not reveal further abnormalities.

The owner elected for hypophysectomy. In the week before presentation for surgery the dog developed more marked PUPD, was markedly glucosuric and was hyperglycemic with a starved blood glucose of 502 mg/dL (RI 64.8‐126.0 mg/dL; 27.9 mmol/L; RI 3.6‐7 mmol/L), consistent with a diagnosis of DM. The dog was prescribed 0.5 U/kg subcutaneous insulin lente (Caninsulin, MSD Animal Health UK Limited, UK) every 12 hours.

The dog was subsequently referred to another center (Royal Veterinary College, Small Animal Referral Hospital) for transsphenoidal hypophysectomy. Preoperative testing confirmed the previously noted hematologic and biochemical findings (see Supplemental Tables 1 and 2). Fructosamine concentration was above the reference range at 468 μmol/L (RI 177‐314 μmol/L). Preoperative serum IGF‐1 was 1558 ng/mL.

A full general anesthetic protocol is available in Supplementary Information. Standard anesthetic monitoring was performed. A bilateral caudal maxillary nerve block was performed with bupivacaine hydrochloride (0.35 mg/kg; AstraZeneca, UK) using a transcutaneous subzygomatic approach, with the needle directed between the caudal aspect of the maxilla and the cranial margin of the mandibular ramus, below the rostroventral border of the zygomatic arch. Intraoperative medications included amoxicillin‐clavulanic acid (20 mg/kg IV; GlaxoSmithKline, UK) every 2 hours, intravenous fluid therapy (Hartmann's solution, 3 mL/kg/h; Vetivex 11, Dechra, UK), neutral insulin in 0.9% NaCl (50‐100 mIU/kg/h IV; Actrapid, Novo Nordisk, UK) and remifentanil hydrochloride (0.1‐0.2 mcg/kg/min IV; Ultiva, Aspen Pharma Trading, Ireland).

Transsphenoidal hypophysectomy was performed largely as previously described, with the additional use of a surgical head clamp (Brainsight, Rogue Research, Canada) as reported in cats for rigid head positioning.^5^, ^6^ The entirety of the grossly visible cream colored pituitary gland was extirpated using a combination of neurosurgical forceps, probes and gentle suction. The pituitary fossa was inspected after pituitary gland removal using a 2.7 mm rigid endoscope (Hopkins Forward‐Oblique Telescope 30°, Karl‐Stortz, Slough, UK) to confirm that there were no macroscopically visible remnants. Closure was performed as previously described (Supplemental Information). Postoperative CT imaging of the head revealed a 5.2 mm × 8.9 mm defect in the basisphenoid bone, with an air‐filled cavity within the pituitary fossa occupying the region of the excised pituitary gland (Figure 2).

Postoperatively the dog made a rapid recovery without complications or neurological deficits. Insulin and hydrocortisone infusions were discontinued 12 hours postoperatively and oral hormone supplementation was instituted (0.5 mg/kg hydrocortisone PO BID, Accord Healthcare Limited, UK) 20 μg/kg levothyroxine PO SID (Thyforon, Dechra Veterinary Products, UK) at the same time voluntary food intake commenced. Application of desmopressin acetate (5 mcg; Aspire Pharma Limited, UK) into the conjunctival sac of alternate eyes was continued every 8 hours. Additionally, 20 mg/kg amoxicillin‐clavulanic acid (Noroclav, Norbrook Laboratories Ltd, UK) was administered every 12 hours orally for 2 weeks postoperatively. Serum sodium increased within the first 12 hours following surgery (153 mmol/L immediately postoperatively, reaching a peak of 177 mmol/L; RI 140‐153 mmol/L). This sodium imbalance was corrected over 6 hours with provision of oral free water which the dog was able to ingest voluntarily, as well as intravenous fluid therapy with 0.45% NaCl (Velit Biopharma S.R.L, Italy). No signs of neurological disease were noted in association with these acute sodium imbalances. The dog had variable glycemic control following surgery with episodes of hyperglycemia following meals with correction to normoglycemia without exogenous insulin therapy.

**FIGURE 3:**
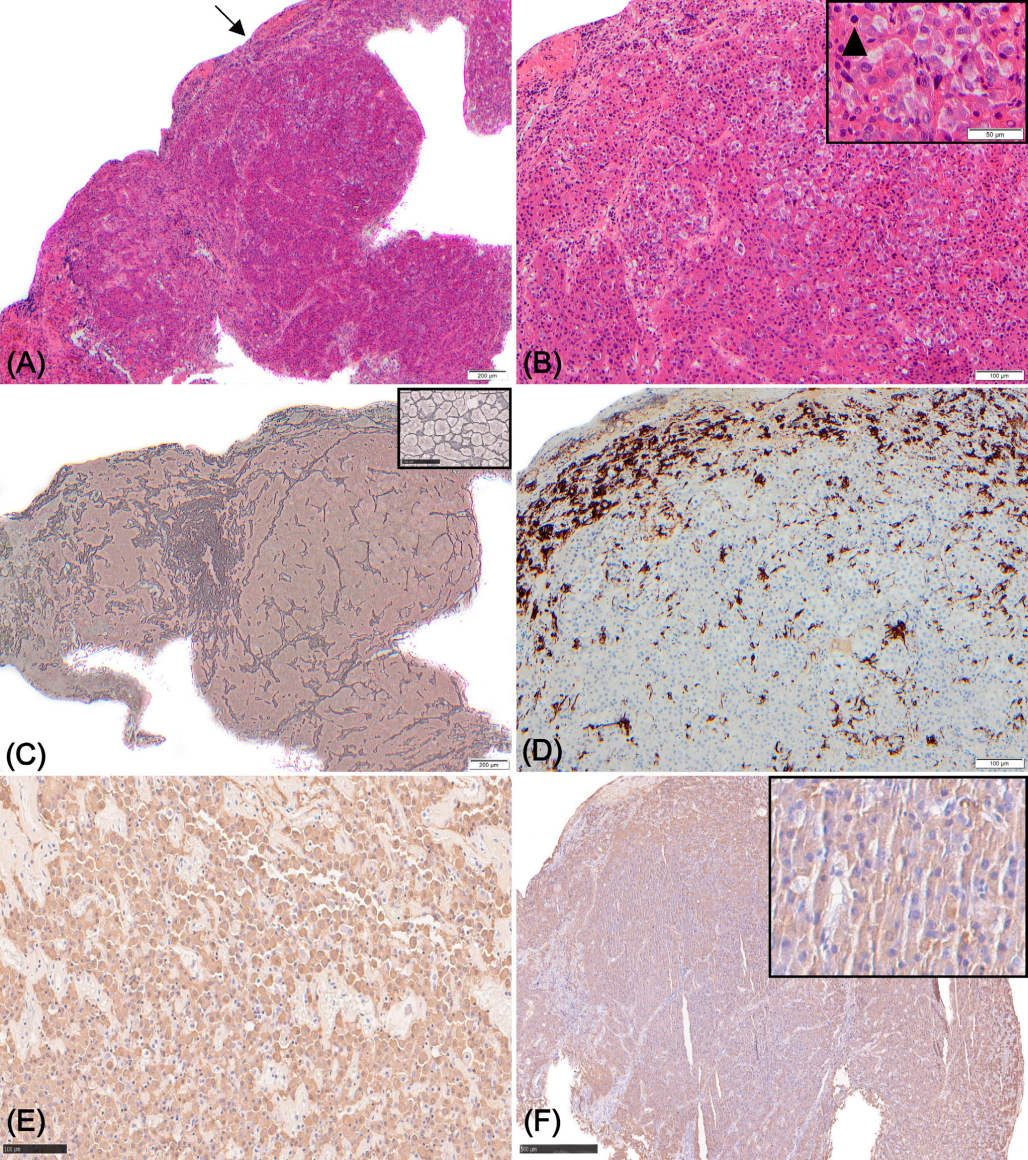
Sections through the excised pituitary neoplasm. (A) H&E. Expanding the pars distalis and compressing the adjacent nonneoplastic parenchyma (arrow) is an unencapsulated poorly demarcated neuroendocrine neoplasm. (B) H&E. Higher magnification of the densely cellular acidophilic neuroendocrine neoplasm, which is composed of polygonal eosinophilic cells exhibiting moderate pleomorphism and occasional mitotic figures (inset; arrowhead). (C) Reticulin. Within the neoplasm there is diffuse loss of the preexisting pars distalis reticular framework compared to a control pituitary gland (inset). (D) Immunohistochemistry (IHC) with antibodies against multicytokeratin (AE1/AE3). There is negative immunostaining of the neoplastic cells. There is strong cytoplasmic immunostaining of the compressed nonneoplastic acidophils (top‐left). (E‐F) IHC with antibodies against growth hormone. (E) Control pars distalis. There is strong cytoplasmic immunostaining of nonneoplastic acidophils. (F) Neoplasm. There is diffuse moderate to strong cytoplasmic immunostaining of the neoplastic cells (inset).

Histopathology of the excised pituitary gland was consistent with a pituitary acidophil neoplasm. Sections from the pituitary gland were immunostained using a rabbit antiporcine GH antibody, as previously described.^7^ Sections from a formalin‐fixed normal pituitary gland from a dog were used as controls. The pituitary tissue was infiltrated with a uniform population of polygonal acidophilic cells which were positively immunostaining for growth hormone and associated with loss of normal reticulin pattern. These findings were consistent with a growth hormone producing pituitary neuroendocrine neoplasm/somatotroph adenoma (Figure 3; full protocol in Supplemental Information).

**FIGURE 4:**
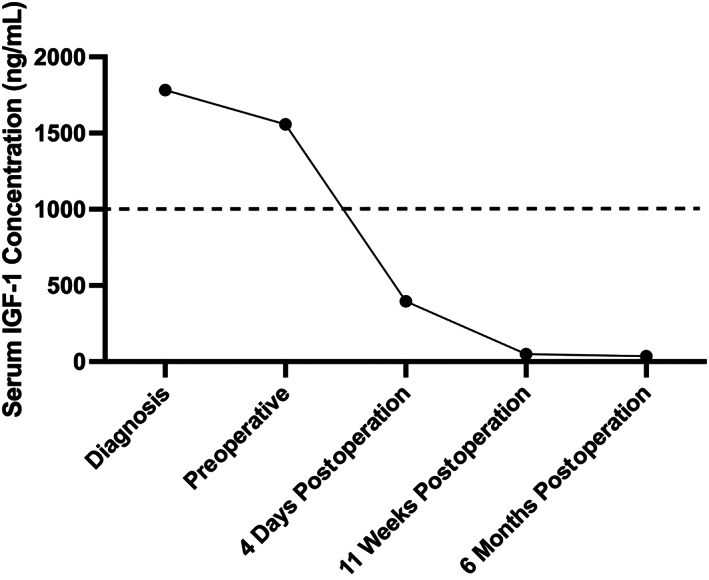
Serum insulin like growth factor‐1 (IGF‐1) concentrations before and after transsphenoidal hypophysectomy. Dashed line indicates IGF‐1 of 1000 ng/mL (reference values are derived from the reference laboratory guidelines: adults of large breeds >200 ng/mL, puppies >500 ng/mL, dwarfism <50 ng/mL, acromegaly >1000 ng/mL; Nationwide Specialist Laboratory Services Ltd, UK).

The dog was discharged 4 days postoperatively, at which time serum IGF‐1 concentration had decreased to 396 ng/mL (Figure 4), then returned 11 weeks later for a follow up appointment. Soon after discharge the dog was persistently hyperglycemic and insulin lente therapy was restarted and gradually increased to 0.3 U/kg twice daily. PUPD had resolved and there were morphological signs of acromegaly resolution, with a return to normal hair length and head appearance (Figure 1), and no inspiratory stertor. There was normalization of the previous mild anemia and serum biochemistry also revealed no abnormalities other than mild hypoglycemia and increased fructosamine (see Supplemental Tables 1 and 2). Serum IGF‐1 concentration was 49 ng/mL, consistent with biochemical remission of hypersomatotropism (Figure 4), and TT4 was 1.32 μg/dL (RI 0.39‐3.42 μg/dL; 17 nmol/L; RI 13‐51 nmol/L). The owners reported that the dog was mildly lethargic and was displaying heat‐seeking behavior. These clinical signs subsequently resolved after an increase of oral levothyroxine dosing to 30 μg/kg once daily. Conjunctival desmopressin was substituted for oral desmopressin acetate (0.1 mg total BID; Aspire Pharma Limited, UK) because of concerns regarding iatrogenic conjunctivitis with the use of desmopressin drops. Six months after surgery serum IGF‐1 concentration was 35 ng/mL (Figure 4).

At 12‐month follow‐up after surgery, the dog was reported to have normal energy levels with no recurrence of previous clinical signs of hypersomatotropism and had normal water intake. The dog was receiving the postoperative medications described above and 0.25 U/kg of subcutaneous insulin lente every 12 hours.

## 3 DISCUSSION

This is a case of a diabetic dog with acromegaly because of a pituitary somatotroph adenoma treated with transsphenoidal hypophysectomy. The reported clinical signs associated with hypersomatotropism, including hair coat changes and inspiratory stertor, resolved by 11 weeks postoperatively. Biochemical remission of hypersomatotropism was achieved and persisted for the 12 months recorded postoperatively. Despite improvements in glycemic control, the dog remained diabetic. As a result, given the clinical and biochemical remission of hypersomatotropism, it is possible that diabetes mellitus developed independently from acromegaly in this dog or irreversible pancreatic beta cell dysfunction occurred secondary to the hypersomatotropism.

Definitive diagnosis of hypersomatotropism was achieved through a combination of compatible clinical signs, increased serum IGF‐1 concentration, evidence of a pituitary mass on advanced imaging and pituitary histology. Pituitary histopathology from acromegalic dogs has rarely been described.^3^, ^8^ Acidophil proliferation accompanied by positive immunostaining for GH in previous cases was similar to our dog and is 1 criteria for the diagnosis of pituitary somatotroph adenomas/neuroendocrine tumor in humans.^9^ In humans with acromegaly, various histologic tumor subtypes have been described and may have some relevance to prognosis in human acromegaly treatment.^10^ Subclassification of tumor types is based on histopathological, immunohistochemical and electron microscopy findings that are currently not utilized in veterinary practice. Pituitary acidophil neoplasms can secrete only GH or prolactin (PRL) concurrently, with the latter being less common and sometimes clinically silent.^10^ Around 30% of humans with acromegaly will have mixed GH and PRL‐secreting pituitary tumors. Cats with hypersomatotropism rarely have mixed tumors and only 1 case report details PRL staining in an acromegalic dog, which was negative.^3^, ^11^, ^12^, ^13^ PRL staining was not performed in this case and might have been useful to exclude a mixed GH and PRL‐secreting tumor although the clinical relevance of such a classification is unknown in dogs. Alongside the pituitary mass, CT imaging also revealed thickened cortices of several limb bones. While this is a subjective finding and should be treated with caution, it could be consistent with thickened bones that have been described in the skull of cats with acromegaly.^14^

Pituitary surgery is the preferred treatment option in human medicine, because of high and rapidly achieved remission rates.^15^ Transsphenoidal hypophysectomy was elected in this dog because of the potential for achieving biochemical and clinical remission of hypersomatotropism, based on findings in humans and cats. Recently, transsphenoidal hypophysectomy has been shown to be an effective treatment option for hypersomatotropism‐induced diabetes mellitus in cats with high rates of diabetic remission (71%‐92%) and median survival times of 853‐1347 days reported.^6^, ^16^, ^17^ Transsphenoidal hypophysectomy is also a well‐described treatment for pituitary‐dependent hyperadrenocorticism and nonfunctional pituitary masses in dogs.^5^, ^18^, ^19^, ^20^

Treatment modalities reported for acromegaly resulting from pituitary tumors in dogs are limited to 2 previous case reports, treated with conventional radiation therapy and medical management.^2^, ^4^ Following radiation therapy in these dogs, both cases had persistent clinical signs of hypersomatotropism and increased serum IGF‐1 concentration, although this subsequently resolved in 1 dog after treatment with pasireotide. There was improvement in glycemic control in both cases, and 1 dog achieved diabetic remission. While newer techniques, such as stereotactic radiation therapy, have been reported for the treatment of hypersomatotropism and diabetes mellitus in cats, diabetic remission rates are not as high as those reported with surgical management, and little information is available regarding changes in serum IGF‐1 concentration following treatment.^21^, ^22^ Serum IGF‐1 concentration in our dog decreased rapidly to normal following surgery (396 ng/mL) and was persistently decreased at 11 weeks postoperatively (49 ng/mL), demonstrating the effectiveness of this procedure in achieving biochemical remission of acromegaly in this dog. As a result, surgical treatment by transsphenoidal hypophysectomy was effective in achieving remission of acromegaly in this dog and can be considered as a first‐line treatment for dogs with pituitary adenoma‐associated growth hormone excess. While there is evidence of treatment benefit associated with medical management,^23^, ^24^, ^25^, ^26^, ^27^ transsphenoidal hypophysectomy provides the additional potential for a curative treatment by removing the hormone‐producing pituitary tumor.

A notable postoperative complication in this case was the development of transient hypernatremia, likely associated with iatrogenic central diabetes insipidus because of the sudden loss of antidiuretic hormone secretion following removal of the posterior pituitary gland.^19^ Increases in serum sodium are documented in dogs and cats after hypophysectomy and careful monitoring of sodium status is an important postoperative consideration.^6^, ^17^, ^28^ Clinicians should be aware of the need for frequent monitoring of fluid and electrolyte balance, supplementation with synthetic desmopressin, provision of adequate oral free water and the use of appropriate intravenous fluid therapy.

A feature of this case was the absence of overt diabetes mellitus at the time of initial diagnosis, its subsequent development and then persistence after hypophysectomy and resolution of hypersomatotropism. This is in contrast to the situation in most cats, where almost all cases of acromegaly are diagnosed as a result of having insulin‐resistant diabetes mellitus.^29^ In dogs, the presence of diabetes mellitus as a result of acromegaly because of mammary GH production is also variable, while the majority of case reports of dogs with pituitary‐dependent acromegaly do appear to be overtly diabetic or have impaired glucose tolerance.^2^, ^3^, ^4^, ^8^, ^30^ PUPD was present before overt diabetes mellitus developed in this dog, although markedly worsened after the onset of DM. PUPD and polyphagia without DM has been noted in 1 dog with pituitary‐dependent acromegaly, although this case was shown to have impaired glucose tolerance.^12^ It is unclear if this was also the case for the dog described in this report.

## CONFLICT OF INTEREST DECLARATION

Authors declare no conflict of interest.

## OFF‐LABEL ANTIMICROBIAL DECLARATION

Authors declare no off‐label use of antimicrobials.

## INSTITUTIONAL ANIMAL CARE AND USE COMMITTEE (IACUC) OR OTHER APPROVAL DECLARATION

Authors declare no IACUC or other approval was needed.

## HUMAN ETHICS APPROVAL DECLARATION

Authors declare human ethics approval was not needed for this study.

## Supporting information


**Data S1.** Supporting information.

## References

[1] Gouvêa FN , Pennacchi CS , Assaf ND , et al. Acromegaly in dogs and cats. Ann Endocrinol. 2021;82(2):107‐111. 10.1016/j.ando.2021.03.002 33727117

[2] Zublena F , Tamborini A , Mooney CT , et al. Radiotherapy and pasireotide treatment of a growth hormone producing pituitary tumor in a diabetic dog. Can Vet J. 2018;59(10):1089‐1093. 30510314 PMC6135304

[3] Van Keulen LJM , Wesdorp JL , Kooistra HS . Diabetes mellitus in a dog with a growth hormone‐producing acidophilic adenoma of the adenohypophysis. Vet Pathol. 1996;33(4):451‐453. 8817849 10.1177/030098589603300417

[4] Reusch C , Burkhardt WA , Meier VS , et al. Acromegaly due to a pituitary tumor in a dog—diagnosis, therapy and long‐term follow‐up. Schweiz Arch Tierheilkd. 2019;161(5):319‐327. 31064738 10.17236/sat00208

[5] Meij BP , Voorhout G , Van den Ingh TS , et al. Transsphenoidal hypophysectomy in beagle dogs: evaluation of a microsurgical technique. Vet Surg. 1997;26(4):295‐309. 9232788 10.1111/j.1532-950x.1997.tb01502.x

[6] Fenn J , Kenny PJ , Scudder CJ , et al. Efficacy of hypophysectomy for the treatment of hypersomatotropism‐induced diabetes mellitus in 68 cats. J Vet Intern Med. 2021;35(2):823‐833. 33624865 10.1111/jvim.16080 PMC7995378

[7] Scudder CJ , Mirczuk SM , Richardson KM , et al. Pituitary pathology and gene expression in acromegalic cats. J Endocr Soc. 2019;3(1):181‐200. 30620005 10.1210/js.2018-00226 PMC6316999

[8] Fracassi F , Gandini G , Diana A , et al. Acromegaly due to a somatroph adenoma in a dog. Domest Anim Endocrinol. 2007;32(1):43‐54. 16472961 10.1016/j.domaniend.2005.12.009

[9] Asa SL , Mete O , Perry A , Osamura RY . Overview of the 2022 WHO classification of pituitary tumors. Endocr Pathol. 2022;33(1):6‐26. 35291028 10.1007/s12022-022-09703-7

[10] Syro LV , Rotondo F , Serna CA , Ortiz LD , Kovacs K . Pathology of GH‐producing pituitary adenomas and GH cell hyperplasia of the pituitary. Pituitary. 2017;20(1):84‐92. 27586499 10.1007/s11102-016-0748-8

[11] Sharman M , Fitzgerald L , Kiupel M . Concurrent somatotroph and plurihormonal pituitary adenomas in a cat. J Feline Med Surg. 2013;15(10):945‐952. 23553410 10.1177/1098612X13483461 PMC11383157

[12] Sanders K , Galac S , Meij BP . Pituitary tumour types in dogs and cats. Vet J. 2021;270:105623. 33641809 10.1016/j.tvjl.2021.105623

[13] Meij BP , Van Der Vlugt‐Meijer RH , Van Den Ingh TSGAM , et al. Somatotroph and corticotroph pituitary adenoma (double adenoma) in a cat with diabetes mellitus and hyperadrenocorticism. J Comp Pathol. 2004;130(2–3):209‐215. 15003481 10.1016/j.jcpa.2003.09.004

[14] Lamb CR , Ciasca TC , Mantis P , et al. Computed tomographic signs of acromegaly in 68 diabetic cats with hypersomatotropism. J Feline Med Surg. 2014;16:99‐108. 23847300 10.1177/1098612X13497212 PMC11383125

[15] Katznelson L , Laws ER , Melmed S , et al. Acromegaly: an endocrine society clinical practice guideline. J Clin Endocrinol Metab. 2014;99:3933‐3951. 25356808 10.1210/jc.2014-2700

[16] Meij BP , Auriemma E , Grinwis G , Buijtels JJCWM , Kooistra HS . Successful treatment of acromegaly in a diabetic cat with transsphenoidal hypophysectomy. J Feline Med Surg. 2010;12(5):406‐410. 20417901 10.1016/j.jfms.2010.03.014 PMC11318759

[17] van Bokhorst KL , Galac S , Kooistra HS , et al. Evaluation of hypophysectomy for treatment of hypersomatotropism in 25 cats. J Vet Intern Med. 2021;35(2):834‐842. 33621385 10.1111/jvim.16047 PMC7995432

[18] Hanson JM , van 't Hooft HM , Voorhout G , et al. Efficacy of transsphenoidal hypophysectomy in treatment of dogs with pituitary‐dependent hyperadrenocorticism. J Vet Intern Med. 2005;19:687‐694. 16231713 10.1892/0891-6640(2005)19[687:eothit]2.0.co;2

[19] Owen TJ , Martin LG , Chen AV . Transsphenoidal surgery for pituitary tumors and other sellar masses. Vet Clin North Am Small Anim Pract. 2018;48(1):129‐151. 29056398 10.1016/j.cvsm.2017.08.006

[20] Hyde BR , Martin LG , Chen AV . Clinical characteristics and outcome in 15 dogs treated with transsphenoidal hypophysectomy for nonfunctional sellar masses. Vet Surg. 2023;52(1):69‐80. 36416123 10.1111/vsu.13917 PMC10100401

[21] Wormhoudt TL , Boss MK , Lunn K , et al. Stereotactic radiation therapy for the treatment of functional pituitary adenomas associated with feline acromegaly. J Vet Intern Med. 2018;32(4):1383‐1391. 29782043 10.1111/jvim.15212 PMC6060317

[22] Watson‐Skaggs ML , Gieger TL , Yoshikawa H , Nolan MW . Endocrine response and outcome in 14 cats with insulin resistance and acromegaly treated with stereotactic radiosurgery (17 Gy). Am J Vet Res. 2022;83(1):64‐71. 10.2460/ajvr.21.08.0122 34773702

[23] Ogedegbe OJ , Cheema AY , Khan MA , et al. A comprehensive review of four clinical practice guidelines of acromegaly. Cureus. 2022;14(9):1‐8. 10.7759/cureus.28722 PMC9453869 36105896

[24] Scudder CJ , Gostelow R , Forcada Y , Schmid HA , Church D , Niessen SJM . Pasireotide for the medical management of feline hypersomatotropism. J Vet Intern Med. 2015;29(4):1074‐1080. 25945588 10.1111/jvim.12608 PMC4895359

[25] Gostelow R , Scudder C , Keyte S , et al. Pasireotide long‐acting release treatment for diabetic cats with underlying hypersomatotropism. J Vet Intern Med. 2017;31(2):355‐364. 28145031 10.1111/jvim.14662 PMC5354018

[26] Miceli DD , García JD , Pompili GA , et al. Cabergoline treatment in cats with diabetes mellitus and hypersomatotropism. J Feline Med Surg. 2022;24(12):1238‐1244. 35133181 10.1177/1098612X221074924 PMC10812327

[27] Scudder CJ , Hazuchova K , Gostelow R , et al. Pilot study assessing the use of cabergoline for the treatment of cats with hypersomatotropism and diabetes mellitus. J Feline Med Surg. 2021;23(2):131‐137. 32684121 10.1177/1098612X20933213 PMC10741349

[28] Meij BP , Voorhout G , Van Den Ingh TSGAM , et al. Results of transsphenoidal hypophysectomy in 52 dogs with pituitary‐dependent hyperadrenocorticism. Vet Surg. 1998;27(3):246‐261. 9605236 10.1111/j.1532-950x.1998.tb00123.x

[29] Niessen SJM , Forcada Y , Mantis P , et al. Studying cat (*Felis catus*) diabetes: beware of the acromegalic imposter. PloS One. 2015;10(5):e0127794. 26023776 10.1371/journal.pone.0127794 PMC4449218

[30] Fracassi F , Zagnoli L , Rosenberg D , Furlanello T , Caldin M . Spontaneous acromegaly: a retrospective case control study in German shepherd dogs. Vet J. 2014;202(1):69‐75. 24986315 10.1016/j.tvjl.2014.06.004

