# Supplementary material for: Transsphenoidal hypophysectomy for the treatment of hypersomatotropism secondary to a pituitary somatotroph adenoma in a dog
Source: J Vet Intern Med. 2023 Nov 2;38(1):351–7. doi: 10.1111/jvim.16929 (PMC10800194; doi:10.1111/jvim.16929)
Supplement: Supplementary file 1 — Data S1. Supporting information. [file JVIM-38-351-s001.pdf]

Supplemental Table 1: Hematology Results

Parameter	Diagnosis	Pre-op	11 weeks post-op	6 months post-op	Reference Interval
HCT (%)	**32.4**	**33.3**	42.5	43.0	37 - 55
RBC (x10^12/L)	**4.61**	**4.68**	6.32	6.82	5.5 - 8.5
HGB (g/dL)	**11.6**	**11.5**	13.9	14.2	12 - 18
MCV (fL)	70.2	71.1	67.2	63.0	60 - 77
MCHC (g/dL)	35.9	34.5	32.7	33.1	32 - 36
Platelets (x10^9/L)	550	604	368	357	150 - 900
WBC (x10^9/L)	10.12	11.06	11.65	11.68	6 - 17.1
Neutrophils (x10^9/L)	6.39	8.63	10.25	7.63	3 - 11.5
Lymphocytes (x10^9/L)	3.01	1.99	**0.93**	2.55	1 - 4.8
Monocytes (x10^9/L)	0.48	0.22	0.35	1.15	0.15 - 1.5
Eosinophils (x10^9/L)	0.19	0.22	0.12	0.30	0 - 1.3
Basophils (x10^9/L)	0.02	0.00	0.00	0.05	0 - 0

HCT, hematocrit; RBC, Red Blood Cell concentration; HGB, hemoglobin; MCV, mean cell volume; MCHC, mean cell hemoglobin concentration.

Supplemental Table 2: Serum biochemistry results

Parameter	Diagnosis	Pre-op	11 weeks post-op	6 months post-op	Reference Interval
Total protein (g/L)	63.4	62.9	56.4	59.0	54.9 - 75.3
Albumin (g/L)	29.3	28.2	29.0	29.0	26.3 - 38.2
Globulin (g/L)	34.1	34.0	27.4	30	23.4 - 42.2
Sodium (meq/L)	145.8	137.3	145.4	149	135 - 155
Potassium (meq/L)	5.14	5.11	4.09	3.9	3.6 - 5.6
Chloride (meq/L)	110.1	**99.8**	109.4	110	100 - 116
Total calcium (mg/dL; mmol/L)	10.54 (2.63)	10.50 (2.62)	9.54 (2.38)	9.34 (2.33)	8.74 - 11.18 (2.18 - 2.79)
Phosphorus (mg/dL; mmol/L)	4.64 (1.50)	4.33 (1.40)	**1.73 (0.56)**	3.22 (1.04)	2.47 - 4.95 (0.8 - 1.6)
Urea (mg/dL; mmol/L)	8.90 (3.18)	13.73 (4.9)	20.73 (7.4)	19.05 (6.8)	8.68 - 28.29 (3.1 - 10.1)
Creatinine (mg/dL; umol/L)	0.85 (75.4)	0.79 (70)	0.98 (79)	1.03 (91)	0.23 - 1.63 (20 - 144.5)
Cholesterol (mg/dL; mmol/L)	**487.2 (12.6)**	**942.4 (24.37)**	151.6 (3.92)	140.4 (3.63)	123.7 - 239.6 (3.2 - 6.2)

Total bilirubin (mg/dL; umol/L)	0.14 (2.43)	0.16 (2.7)	0.19 (3.3)	0.18 (3.0)	0.01 - 0.25 (0.1 - 4.2)
ALT (U/L)	39.0	39.6	37.2	23	19.8 - 124
ALP (U/L)	60.0	78	61	64	0 - 130
Glucose (mg/dL; mmol/L)	109.8 (6.1)	**379.8 (21.1)**	**63.0 (3.5)**	87.7 (4.9)	64.8 - 126 (3.6 - 7.0)
Fructosamine (umol/L)	-	**467.6**	414.7	–	177 - 314
Total thyroxine (µg/dL; nmol/L)	1.4 (18.3)	-	1.3 (17.0)	**4.5 (59.0)**	0.4 - 3.4 (5.0 - 44.0)

Bracketed values denote system international (SI) units; values in bold are outside the reference interval; ALT, alanine aminotransferase; ALP, alkaline phosphatase.

Supplemental Information

Anesthetic Protocol

The dog was pre-medicated intramuscularly with methadone (0.2 mg/kg; Synthadon, AnimalCare Limited, UK) and dexmedetomidine (0.0025 mg/kg; Dexdomitor, Zoetis, UK). An intravenous catheter was then aseptically placed into a cephalic vein (22G, Jelco, UK), anesthesia induced using propofol (3.6 mg/kg; PropoFlo Plus, Zoetis, UK) and orotracheal intubation performed using an armoured endotracheal tube (7.0 mm ID, AceVet Limited, UK). A thickened larynx and slightly elongated soft palate were noted on airway examination.

Anesthesia was maintained using sevoflurane (SevoFlo; Zoetis, UK) in 100% oxygen, and standard monitoring with pulse oximetry, electrocardiography, oscillometric, Doppler and invasive blood pressure, rectal temperature, fraction inspired oxygen and sevoflurane, end-tidal sevoflurane, end-tidal carbon dioxide, spirometry, airway pressures and tidal volume and capnography were performed. Serial arterial blood gas monitoring revealed a respiratory acidosis which was mitigated using pressure-controlled intermittent partial pressure ventilation.

The dog was instrumented aseptically with a long-stay catheter (4.5 Fr, 12.5 cm; Mila International, UK) in the medial saphenous vein for venous access and an intra-arterial catheter (22G; Jelco, UK) placed in the dorsal metatarsal artery for invasive blood pressure monitoring and arterial blood sampling. A bilateral caudal maxillary nerve block was performed with bupivacaine hydrochloride (0.35 mg/kg; AstraZeneca, UK) to desensitise the maxilla. Intravenous amoxicillin-clavulanic acid (20 mg/kg; GlaxoSmithKline, UK) was given every two hours for prophylactic antibiosis.

Intravenous fluid therapy was instituted using Hartmann's solution (3 ml/kg/hr; Vetivex 11, Dechra, UK) and intraoperative hyperglycemia was treated with an infusion of neutral insulin in 0.9% NaCl (50 – 100 mIU/kg/hr; Actrapid, Novo Nordisk, UK) to good effect. A continuous rate infusion of

remifentanil hydrochloride (0.1 – 0.2 mcg/kg/min; Ultiva, Aspen Pharma Trading, Ireland) was used to provide additional antinociception and volatile agent sparing effect. In brief, after soft palate incision and creation of a 5.0 mm wide burr hole in the basisphenoid bone, the ventral dura overlying the pituitary gland was visualised and sharply incised peripherally to reveal the pituitary mass. At the time of surgical manipulation of the pituitary gland an intravenous hydrocortisone infusion (0.5 mg/kg/hr IV; Solu-Cortef, Pfizer, UK) was delivered and desmopressin acetate (5 mcg; Aspire Pharma Limited, UK) was administered sublingually at this time. Closure was performed as previously described, with a two-layer closure of the soft palate and routine closure of the temporal muscle and skin incisions that were needed for the head clamp.

A long-stay catheter (4.5 Fr, 12.5 cm; Mila International, UK) was placed in the jugular vein after postoperative computed tomography was performed to replace the long-stay catheter in the medial saphenous vein. Regurgitation occurred prior to recovery from anesthesia, so suctioning of the oesophagus was performed and maropitant (1 mg/kg; Prevomax, Dechra, UK) and omeprazole (1 mg/kg IV; Mylan, UK) were administered intravenously. Recovery otherwise proceeded without complication.

GH Staining Protocol

Tissue was fixed in 10% formalin at room temperature for at least 48 hours before embedding in paraffin wax. 4 micrometre sections were cut on adhesive polysine slides (10219280, Fisherscientific, Massachusetts, United States). Slides were heated at 60°C for 15 minutes, then dewaxed in two changes of xylene for 5 minutes and then industrial methylated spirit (IMS) for 2 minutes. Endogenous peroxidase activity was blocked by placing slides into 2% hydrogen peroxide (H_2O_2) for 5 minutes. Slides were placed into IMS solution for 2 minutes and then transferred to running water. Antigen retrieval was performed in a pressure cooker at high pressure for 10 minutes in 2 mM citric

acid and 9 mM trisodium citrate dehydrate, pH 6.0 (H3300, Vector, Stuttgart, Germany). Slides were cooled slowly by running water into the vessel containing the slides.

A hydrophobic pen (H-4000, Vector, Stuttgart, Germany) was used to outline specimens, and specimens were covered in wash buffer (S3006, Dako, California, United States). Primary antibodies (rabbit anti-porcine GH, National Hormone & Peptide Program, Harbor-UCLA Medical Center, California, USA) were diluted to the appropriate concentration in emerald-green diluent (936B, Sigma-Aldrich, Missouri, United States). Immunostaining was performed using an automatic staining machine (DAKO autostainer, California, United States), using the Super Sensitive™ Polymer HRP Kit (QD400-GP, Biogenex, California, United States). Primary antibodies were incubated for 40 minutes at room temperature (unless specified otherwise). Slides were washed before applying super enhancer for 20 minutes then washed and SS-label incubated for 30 minutes. Slides were washed before Appling 3.3'diaminobenzidine (DAB) reagent for 10 minutes. The antigen-antibody complex was visualized with DAB (Vector, SK-4100 or QD400-GP, Biogenex, California, United States).

After staining slides were removed from the DAKO machine (DAKO autostainer, California, United States), and counterstaining was performed. Wash steps involved placing the slides in running water for 1 minute. Slides were incubated in hematoxylin for 5 minutes and washed. Slides were dipped in acid alcohol solution 5 times then washed. Slides were incubated in Scott's solution for 3 minutes and washed. Slides were dehydrated using 3 changes of IMS for two minutes then 3 changes of xylene for 2 minutes. Slides were mounted and coverslips applied. Slides were dried overnight before scanning on a Nanozoomer 210 (Hamamatsu Photonics, Shizuoka, Japan).

For negative controls antibody diluent without any primary antibody was incubated and no specific immunoreactivity was detected in these tissue sections.
